# Characteristics and Transition of Sleep–Wake Rhythm in Nursery School Children: The Importance of Nocturnal Sleep

**DOI:** 10.3390/clockssleep6040045

**Published:** 2024-11-12

**Authors:** Takehiro Hasegawa, Shozo Murata, Tatsuo Kagimura, Kaoru Omae, Akiko Tanaka, Kaori Takahashi, Mika Narusawa, Yukuo Konishi, Kentaro Oniki, Teruhisa Miike

**Affiliations:** 1 Art Childcare Corporation, 3F, 1-3-10 Higashi-Shinagawa, Shinagawa-ku, Tokyo 140-0002, Japan; kuha485@gmail.com (T.H.); murata@the0123.com (S.M.); tanaka-akk@the0123child.co.jp (A.T.); takahashi-kor@the0123child.co.jp (K.T.); narusawa@the0123child.co.jp (M.N.); 2 Center for Baby Science, Doshisha University, Kyoto 619-0225, Japan; drkonisi@dream.ocn.ne.jp; 3 Translational Research Center for Medical Innovation (TRI), Foundation for Biomedical Research and Innovation at Kobe, 1-5-4 Minatojima-minamimachi, Chuo-ku, Kobe 650-0047, Japan; kagimura@fbri.org (T.K.); omaekaoru@gmail.com (K.O.); 4 Division of Pharmacology and Therapeutics, Graduate School of Pharmaceutical Sciences, Kumamoto University, Kumamoto 862-0973, Japan; oniken@kumamoto-u.ac.jp; 5 Department of Child Development, Kumamoto University, Kumamoto 860-8556, Japan

**Keywords:** sleep–wake rhythm, nursery children, nighttime basic sleep duration, catch-up sleep, social jet lag

## Abstract

In this study, we investigated the sleep–wake rhythm of nursery school children with the aim of supporting their health and mental/physical development. We analyzed 4881 children from infancy to 6 years of age, using 2 week sleep tables recorded by their guardians. The tables contained night bedtimes, wake times, nighttime/daytime sleep duration, and the differences in these between weekdays and weekends. The total sleep decrement of children with increasing age is attributed to a decrease in daytime sleep, while nighttime sleep duration remains almost unchanged at about 10 h, which is, therefore, referred to as the nighttime basic sleep duration (NBSD). Although bedtime stabilizes at around 9:30 p.m. by the age of 2, wake-up times tend to be before 7 a.m., which results in sleep insufficiency during weekdays. This lack of sleep is compensated for by long naps on weekdays and by catching up on sleep on weekend mornings, which may contribute to future social jet lag. Guardians are encouraged to know their children’s exact NBSD and set an appropriate bedtime to be maintained on weekdays. This helps to prevent sleep debt and fosters a consistent daily rhythm of waking up at the same time both on weekdays and weekends. These conditions are believed to support mental/physical development and school and social adaptation.

## 1. Introduction

It is widely recognized that adequate sleep is especially important for children. An excellent report has been published on the appropriate duration of sleep needed per day for children, and the authors also emphasize the importance of “sleep on a regular basis,” which is associated with better health outcomes, including the following factors: improved attention, behavior, learning, memory, emotional regulation, quality of life, and mental and physical health [1]. Discussions about children’s sleep problems have primarily focused on “insufficient sleep” by examining the total sleep duration per day, with few reports focusing on nighttime sleep duration or chronobiological rhythms. However, recent reports indicate that nighttime sleep, as part of the total amount of sleep, has a significant impact on children’s mental and physical development [2,3,4]. This finding has led to major advancements in future research on children’s sleep problems. Incidentally, reports on infants’ nighttime sleep duration indicate that a continuous sleep rhythm of about 8 to 12 h is typically established by 6 to 7 months of age [5,6]. One of the authors has also been conducting ongoing research into the importance of sleep–wake rhythms in the lives of infants and young children, reporting that ensuring an adequate nighttime sleep duration (called nighttime basic sleep duration (NBSD)) and maintaining regularity in daily rhythms are particularly important for children’s mental and physical development [7].

Nowadays, the nocturnal lifestyle of modern people working or acting late at night under bright lights has become daily routine not only in Japan, but also in other countries around the world. The sleep onset time for children involved in such a late-night lifestyle tends to become delayed, often approaching midnight. On the other hand, such parents and children are required to wake up between 6 and 7 o’clock in the morning due to present-day school and social life. Consequently, chronic sleep deprivation and its accumulation are a more serious concern for children than for their parents.

For example, in Japan, the most common bedtime for infants was around 8 p.m. in the 1960s; however, currently, 10–30% of children fall asleep after 10 p.m. [8], representing a shift of more than 2 h. A survey comparing the percentage of Japanese children who went to bed after 10 p.m. in 1980, 1990, and 2000 reported the following [9]: a marked increase was observed in children aged 18 months (25% → 38% → 55%), 2-year-olds (29% → 41% → 59%), 3-year-olds (22% → 36% → 52%), 4-year-olds (13% → 23% → 39%), and 5–6-year-olds (10% → 17% → 40%). These data show that children’s lifestyle rhythms are becoming more nocturnal over the years. Meanwhile, weekday wake-up times have remained roughly between 6:50 and 7:00 p.m. [9], with no major change. Wake-up times have remained the same for decades, resulting in continuous sleep deprivation and the potential for “sleep debt.” A modern nocturnal lifestyle is widespread globally, affecting children by forcing them to sleep late and wake up early, thereby deteriorating their sleep environment.

According to previous research, sleep issues in modern society have garnered increasing attention, and media coverage has grown. However, most of this coverage focuses on adult sleep issues, mainly concerning sleep duration, with little consideration given to children’s sleep issues and human biological rhythms.

It has long been said that getting a good night’s sleep is important for children’s growth. A study warned that small amounts of sleep restriction in children could pose a risk of unpredictable disorders in their emotional and cognitive functioning [10]. Particularly in childhood, sleep is associated with the brain (e.g., development, growth, repair of damage, protection of function, and further evolution), indicating that adequate sleep is much more important for children than for adults. As mentioned above, a certain amount of longer nighttime sleep is better for children’s growth. Additionally, it is also known that an appropriate biological rhythm is quite important for children’s balanced mental/physical development, because the sleep/wake rhythm is controlled by the biological clock. Therefore, sleep affects children emotionally and behaviorally [11], and sleep disorders may lead to anxiety, hyperactivity, aggression, and ultimately negative effects on mental/physical development [1,7].

Additionally, children’s sleep problems are commonly found, and one study has reported that chronic sleep deprivation may shift their biological clock and could even lead to neuronal loss [12]. Furthermore, the circadian rhythm’s biological clock not only controls the sleep/wake rhythm, but also the so-called life support systems (e.g., the autonomic nervous system, hormone secretion, thermoregulation, energy metabolism, the immune system, coordinated movement, and the maintenance of brain function balance) [13,14]. Therefore, it is important to consider children’s daily life rhythms in order to support their health and physical/mental development [15].

Based on the above information, it is clear that understanding the actual sleep/wake rhythms of children is necessary for monitoring the physical and mental growth of nursery school children and their future health. To achieve this, we planned to investigate and analyze daily life rhythms using two weeks of sleep records.

## 2. Results

### 2.1. Night Bedtime and Morning Wake Time

**Figure 1:**
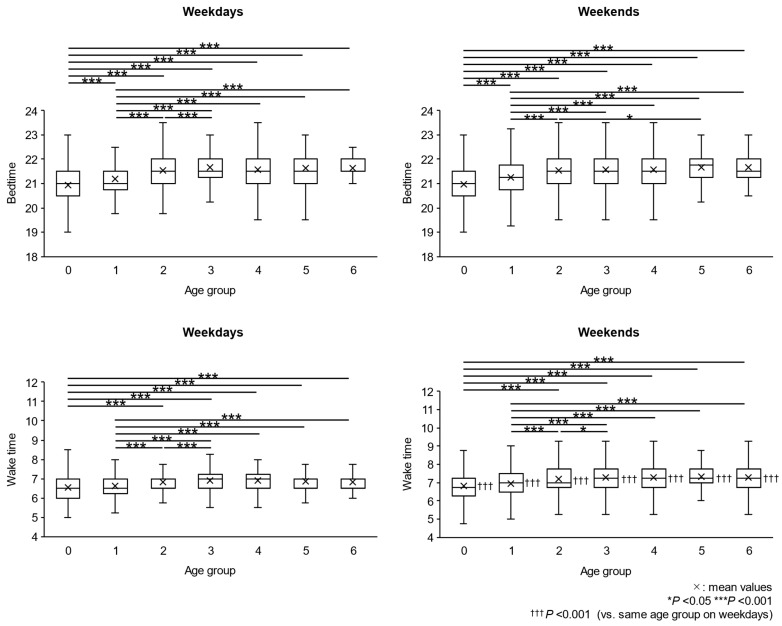
Night bedtime and morning wake time of each age group on weekdays and weekends. The bottom and top edges of the boxes represent the 25th and 75th percentile of the data, respectively. The lines in the boxes represent the median of the data. The lower and upper ends of the error bars represent the minimum and maximum values, excluding outliers, respectively.

Night bedtime and morning wake time for nocturnal sleep on weekdays and weekends are shown by age groups in Figure 1. Night bedtime increased with increasing age from 0 to 6 years old on both weekdays and weekends (trend test: *p* < 0.001 for each). Morning wake time increased with age from 0 to 6 years old on both weekdays and weekends (trend test: *p* < 0.001 for each). A comparison of the average night bedtime on weekdays and weekends for each age group showed no significant differences. On the other hand, when comparing the average morning wake time on weekdays and weekends for each age group, the average morning wake time was significantly later on weekends for each age group.

### 2.2. Nocturnal Sleep Duration and Daytime Sleep Duration

**Figure 2:**
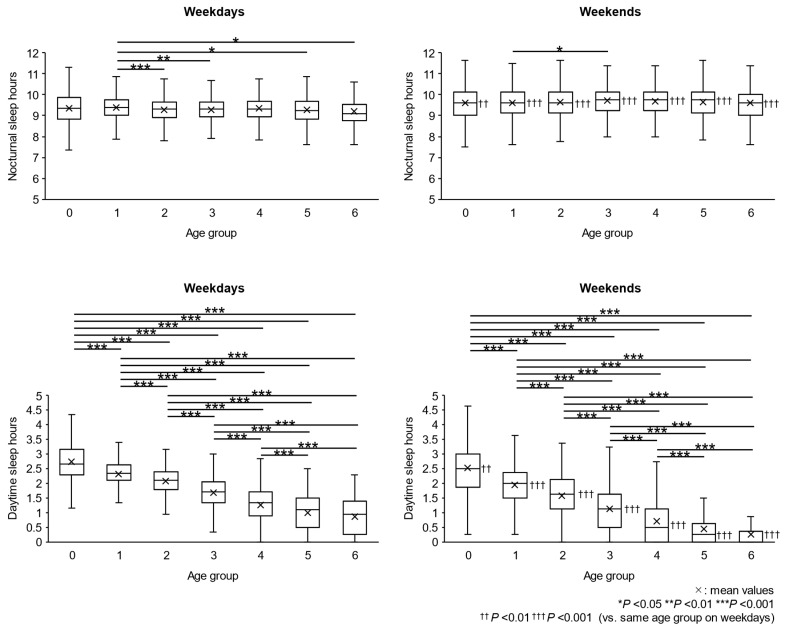
Nocturnal sleep duration and daytime sleep duration of different age groups on weekdays and weekends. The bottom and top edges of the boxes represent the 25th and 75th percentile of the data, respectively. The lines in the boxes represent the median of the data. The lower and upper ends of the error bars represent the minimum and maximum values, excluding outliers, respectively.

Nocturnal and daytime sleep duration on weekdays and weekends are shown by age groups in Figure 2. Nighttime sleep duration did not differ significantly between the age groups on either weekdays or weekends. On the other hand, there were significant differences in daytime sleep duration between the age groups on both weekdays and weekends, with a decrease from groups aged 0 years to 6 years of age. In all age groups, nighttime sleep duration was significantly longer on weekends than on weekdays, while the daytime sleep duration was significantly shorter on weekends than on weekdays.

### 2.3. Relationship Between Night Bedtime and Morning Wake Time

**Figure 3:**
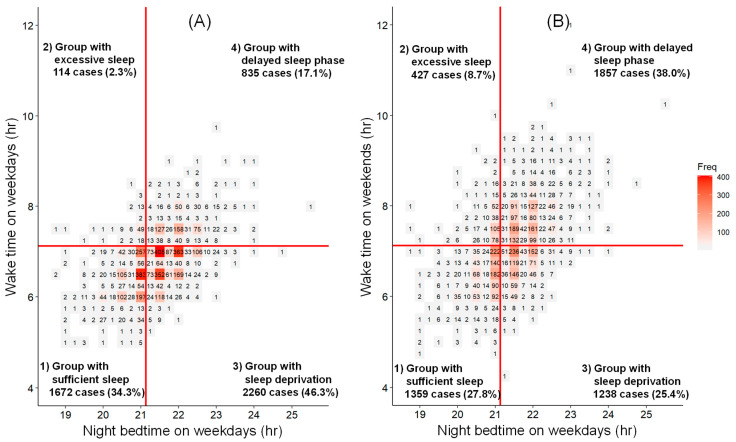
Relationship between bedtime on weekdays and wake time on weekdays and weekends. The graph is a heat map, with the x-axis representing the bedtime of all of the participants on weekdays and the y-axis showing the wake time of all of the participants on weekdays (**A**) and weekends (**B**). The vertical line separates the groups that slept no later than 21:00 and after 21:00, and the horizontal line separates the groups that woke no later than 7:00 and after 7:00. The lower right quarter of the graph suggests a tendency of chronic sleep deprivation, and the upper right quarter suggests a backward shift in life (chronobiological) rhythm.

The relationship between night bedtime and morning wake time on weekdays was studied, and a tendency of later bedtime leading to a later wake time was found (Figure 3A).

The participants were divided into four groups by a bedtime of no later than 21:00 or after 21:00 and a wake time of no later than 7:00 or after 7:00. The four groups were classified as follows: (1) the group with sufficient sleep whose bedtime was no later than 21:00 and wake time was no later than 7:00; (2) the group with excessive sleep whose bedtime was no later than 21:00 and wake time was after 7:00; (3) the group with sleep deprivation whose bedtime was after 21:00 and wake time was no later than 7:00; and (4) the group with a delayed sleep phase whose bedtime was after 21:00 and wake time was after 7:00.

Then, the relationship between bedtime on weekdays and wake time on weekends was studied, wherein 80.3% of the group with sufficient sleep on weekdays remained in the same group on weekends, and 85.1% of the group with excessive sleep on weekdays also remained in the same group on weekends. On the other hand, 48.8% of the group with sleep deprivation on weekdays had a delayed waking time on weekends. It was found that children with a later bedtime on weekdays were apt to wake up later on weekends (Table 1 and Figure 3B).

## 3. Discussion

When evaluating sleep–wake rhythm, it is difficult to understand the entire daily life rhythm of participants only by frequently used bedtime and wake-up time records. In this study, we used 2 week sleep record tables, which enabled an overview of the entire daily life rhythm of the participants [16].

The evaluation of time spent and life rhythm on weekends was a crucial aspect of the study, and sleep records of at least two weekends were required to account for special events, such as trips, that might occur on weekends. On the other hand, it was pointed out that sleep records of children reported by their guardians could be less accurate than those obtained through actigraphy, with parent-reported sleep times being longer by 24 to 45 min compared to actigraphy records [17,18,19]. However, in another study, sleep record tables were considered clinically useful enough, despite acknowledging their shortcomings [20,21]. Indeed, in our previous study, we observed that the actigraphy judged motionless participants as sleeping and recorded longer sleep times than the actual sleep time.

In Japan, it is common for guardians (mainly parents) to share a bedroom with their infants and young children, and it is estimated that such parental observations are relatively more accurate. According to these factors, it is considered that the use of sleep record charts in clinical settings is meaningful enough at this time.

In this study, bedtime and wake-up time gradually shifted after birth, becoming just over 30 and 20 min later by age 2, respectively. On the other hand, almost no changes were observed in these items after that (Figure 1). It is speculated that this phenomenon could be due to the relatively early formation of the sleep–wake circadian rhythm [22,23,24] or the influence of the postnatal living environment [25,26,27], but a clear explanation has not been given.

Night bedtime was not different between weekdays and weekends, while morning wake-up time on weekends was significantly later than that on weekdays. Along with the later wake-up time on weekends, naps on weekends were shorter than those on weekdays. The explanation for this is that the children compensated for nighttime sleep deprivation on weekdays by taking longer naps on weekdays and sleeping longer on weekend mornings [19,28]. It could be suggested that the sleep–wake rhythm on weekends is a suitable index for representing the natural life rhythm for many children. In our data, the total sleep duration on weekends for 6-year-old children, who are expected to have no daytime sleep in preparation for future school life, is approximately 10 h on both weekdays and weekends.

The data showed that the decline in total daily sleep time with age was due to a decrease in daytime sleep time, which began to disappear at age 3, while nighttime sleep time remained constant across all age groups. This average 10-h sleep duration is considered to be required for Japanese children as basically necessary nighttime sleep, so we defined it as “nighttime basic sleep duration (NBSD)” [7].

Similar results have been reported in Switzerland [29], England [30], and Canada [24]. In Switzerland, the average nighttime sleep duration for children aged 1 to 5 years is more than 11 h (11.7 to 11.1 h); in the United Kingdom, that for children aged 1 to 6 years is approximately 11 h; and, in Canadia, that for children aged 6 months to 4 years is 10 h (47.6%) or 11 h (42.8%), respectively. Interestingly, these papers report that total sleep time decreases with age, but nighttime sleep time shows little change. The report from Switzerland [29] found that nighttime sleep duration increased slightly from infancy to 1–3 years of age and then returned to the infant level by 5–6 years of age. This can be interpreted as meaning that there was no significant increase or decrease in the group aged 1–6 years.

Although neither report specifically highlights this almost fixed nighttime sleep duration, the data in these reports commonly show that nighttime sleep duration remains relatively stable from early childhood to 6 years of age. Meanwhile, daytime sleep duration gradually decreases, disappearing by ages 2 to 5 [31], or by age 7 at the latest [32].

The duration of nighttime sleep in the aforementioned countries is generally around 11 h, which is slightly longer than the 10 h reported in this study. A longer sleep time of Caucasian children compared to children in Asian countries was also reported by Mindell et al. in 2010 [33]. Their report mentioned that the average nighttime sleep duration of Japanese children aged 0–3 years was 9 h and 42 min, which is almost the same as the weekend sleep duration reported in this study. The report states that, when comparing the average daily total sleep time of children of the same age group, Japanese children sleep 11 h and 37 min, (11 h and 42 min in this study), which is 100 min less than the 13 h and 17 min of children in New Zealand, the longest recorded averaged daily total sleep time.

It is speculated that Western parenting books recommend that infants fall asleep between 7 and 8 p.m. due to the length of nighttime sleep. The difference in sleep duration may be ascribed to cultural differences, such as bedroom sharing, and differences between races. Furthermore, the difference may be attributed to differences in living environment temperatures between Asia and Western nations, with Asia experiencing comparatively warmer weather and Europe and the United States being located further north. This is because people generally sleep less in summer and more in winter. Specifically, a 1960 survey (of people aged from 16 to 65) conducted by NHK (NIPPON HOSO KYOKAI: Japan Broadcasting Corporation, Tokyo, Japan) on sleep time reported an average sleep time of 8 h and 4 min in summer and 8 h and 40 min in winter.

In this report, children were divided into the following four groups based on an average NBSD of approximately 10 h and their daily life rhythms (Figure 3A,B): (1) group with sufficient sleep, (2) group with excessive sleep, (3) group with sleep deprivation, and (4) group with delayed sleep phase. Group 1 consists of children who fall asleep before 9 p.m. and wake up by 7a.m.; Group 2 consists of children who fall asleep before 9 p.m. but wake up after 7 a.m., indicating that they need more than 10 h of sleep; Group 3 consists of children who fall asleep after 9 p.m. and wake up by 7 a.m., with daily sleep clearly less than 10 h and concerns about a lack of nighttime sleep; and Group 4 consists of a group of children who fall asleep after 9 p.m. and wake up after 7 a.m., indicating that there has been a shift in their daily rhythm backwards. These data show that a number of children need some adjustment in their daily life rhythm.

For example, the children in Group 2 need to have more than 10 h of sleep before waking up in the morning, so they need to get into the habit of falling asleep earlier, such as at 7 or 8 p.m., to avoid sleep loss, while the children in Group 3 need to get into the habit of falling asleep by 9 p.m. It is particularly important to understand the NBSD of each child in all groups, especially in Group 4, and ensure that each NBSD is met by an appropriate time to wake up in the morning, which is necessary for maintaining health, socially well-adjusted behavior, and mental/physical development.

The children who wake up late to compensate for sleep shortage on weekends, primarily those in Group 3 and Group 4, were found to fall asleep later and have insufficient sleep before 7 a.m. (refer to Figure 3A,B).

In addition, some children who wake up late on weekends maintain an unbalanced bedtime and wake time. In a few cases, children who have an almost proper night bedtime, such as 21:00, could not have sufficient sleep because of their early morning wake time, such as 5:00, due to their family schedules. One of the reasons for the later wake time on weekends than weekdays is that the participants are children who attend nursery. A study reported that children who go to nursery because their parents leave home early in the morning for work are more apt to have nocturnal life habits than children who attend kindergarten and have one parent who always stays at home. The situation, however, is estimated to be common not only for children who attend nursery, but also for all children in Japan [34]. The current bedtime for 30% [8] of children in Kizugawa, Japan, has been reported to be later than 22:00. In our study, the bedtime for at least 10% of the participants on weekdays and the bedtime for at least 15% of the participants on weekends was later than 22:00. Such children are required to wake up between 6:00 and 7:00 in the morning in the present-day school and social life, and this decreased sleep time causes chronic sleep deprivation. As mentioned above, even small amounts of sleep restriction in children could cause a risk of unpredictable disorder of their emotional and cognitive functioning [10].

The late morning wake time on weekends, which is usually seen in Japanese society, is also common worldwide, as reported in Hong Kong, Korea, Canada, Norway, the U.S.A., and Australia [28,35,36,37,38,39]. It has been estimated that the daily life rhythm of children is strongly influenced by the daily life rhythm of their family, resulting in sleep deprivation on weekdays, which is compensated for by an extended nap time on weekdays and late morning wake time on weekends [28].

This study showed that sleep duration on weekend nights was statistically longer than on weekdays for all age groups, suggesting that preschool children are persistently slightly sleep deprived on weekdays.

Considering that the weekend sleep duration is more appropriate for children, our data suggest that a sleep duration of at least 9 h and 40 min is necessary every night. In addition, it highlights the importance of sufficiently long nighttime sleep duration [40,41].

Nap-time sleep usually shortens with age and tends to be shorter on weekends than on weekdays. In other words, nighttime sleep duration is longer and nap-time sleep is shorter on weekends. This means that securing individual NBSD leads to shorter nap-time sleep [42]. A study reported that the youngest children who stopped napping were 2 years old [31], and, in our study, the youngest children who stopped napping were approximately 3 years old. Nurseries in Japan are apt to encourage children to take naps, and even children aged 5 or 6 years old usually take naps at nurseries. However, many children aged 5 or 6 years do not take naps at home on weekends, and a study reported that 90% or more of children stop napping at the age of 5 years [32]. This study did not analyze the number of naps children took during the day, but there are excellent papers on the topic. In Japan, children take multiple naps until about 7 months of age, including one in the morning and one in the afternoon (two naps per day), and then settle down to one nap in the afternoon from about 14 months of age [22]. The recommended time for an afternoon nap is between 12:00 and 15:00. In the United States, naps are reported to decrease to two naps per day from approximately 9 to 12 months of age, and to one nap in the afternoon from 15 to 24 months of age [32].

In nurseries, children having insufficient nocturnal sleep can compensate for the shortage by taking naps. However, children aged 5 or 6 years would enter primary school (school starts in April in Japan) 6 months after the study (in September), thus starting a life without daytime sleep. Therefore, the sleep time required for children must be covered by nighttime sleep without compensation by napping. It is important to consider that the sufficient amount of sleep for children should not be satisfied by the total of nighttime sleep plus nap-time sleep, but must be satisfied by appropriate individual NBSD and, if necessary, by napping. A former study reported that all children stop napping by the age of 7 [32], and it is construed that those children cannot take naps at school. Children should stop napping before entering elementary school, and securing NBSD is the required condition to stop napping in Japan. Such a life habit without napping cannot be achieved in a short period of time, but rather needs to be gradually formed into a daily rhythm from an early stage in infancy.

The data in this study prove that some children have insufficient sleep on weekdays or have a habit of waking after 7 a.m. due to a shift in their living hours. Life rhythm is the basic factor in children forming their biological clock, and we must not ignore the number of children whose biological clocks have shifted [7,12].

In preparation for entering elementary school, it is important for children to make a habit of waking up by themselves at 6 to 7 a.m. to ensure that they can attend classes beginning at around 8:00~8:30. To secure NBSD (about 10 h on average in Japan and 10 to 11 h in Western countries) [24,29,30,31] before waking at 6 to 7 a.m., the appropriate sleep onset time falls within the range from 7 to before 9 p.m.

A life rhythm with no difference in bedtimes and wake times between weekdays and weekends and with sufficient sleep secured before 6 or 7 a.m. in the morning is necessary for children to adapt to their school/social life, and it protects their mental and physical health throughout their life.

The results of this study underscore that the timing of sleep during the day is crucial for evaluating children’s sleep, rather than focusing only on the total amount of sleep time. Children are engaged in school and social activities and must accommodate their school schedule when establishing their biological clock. When children establish a well-regulated circadian rhythm, primarily consisting of a sleep–wake rhythm that aligns with their school life, they can lead a healthy school and social life, both physically and mentally. A gap between their biological clock and the schedule of school activities causes social jet lag, which leads to many problems in school life. The greater the jet lag, the more overloaded they feel in keeping up with school life. This will inevitably disrupt their biological clock and affect various aspects of their bodily functions [13,43,44,45].

Recently, it was reported that the biological clock formed in childhood, including fetal and neonatal periods, affects the future development and physical and mental health of children. Thus, the importance of biological clock creation has been discussed [46,47]. The background of the discussion includes several reports indicating that a shifted or disturbed circadian rhythm increases the risks of future health damage. For example, several studies have reported that a disturbed circadian rhythm induces chronic inflammation in the body, potentially shortening the lifespan [48]. Infants who align their life rhythm with the circadian rhythm have a lower risk of needing medical treatment for diseases by the age of 6 years [49]. Additionally, changes in the sleep behavior of young children significantly relate to the changes in their emotional functioning, attentiveness, and physical behavior during the day [10,50,51]. Furthermore, addressing sleep problems is crucial for improving the behaviors of children with neurodevelopmental disorders [52,53,54]. These studies underline the importance of establishing a circadian sleep–wake rhythm in infancy and childhood.

In fact, many of the children with the problems mentioned in the Introduction are included in Groups 2–4, and it is speculated that the disruption of daily rhythms has a negative impact on children’s mental and physical development and emotional balance. Conversely, it has been suggested that these disruptions to daily rhythms indicate variations in children’s vital functions, and that this irregularity in daily rhythms may actually impair the balance of children’s mental and physical development [7]. In addition, in clinical terms, it has been reported that correcting disruptions to daily rhythms can restore balanced mental and physical functions [55].

## 4. Conclusions

Through this study, we would like to highlight/emphasize that establishing a daily rhythm centered around consistent daily routines is a crucial factor for the mental and physical development of children.

It is suggested that both the length of sleep duration and an appropriate sleep time-zone are critical for supporting children’s physical/mental development. Guardians should first determine their child’s exact nighttime basic sleep duration (NBSD) and establish an appropriate bedtime to ensure this NBSD before the required wake-up time in the morning (before 7 a.m. in Japan), thereby preventing the accumulation of sleep deprivation and shifts in daily rhythms, including social jet lag.

We would like to emphasize that maintaining this proper daily rhythm may be essential for ensuring balanced mental/physical development, as well as school and social adaptation, since it helps to synchronize all aspects of the life-sustaining biological clock. Furthermore, these recommended daily rhythms are best introduced before 18 months and 2 years of age, when the sleep–wake central circadian rhythm is almost established in the suprachiasmatic nucleus, which has a positive impact on the mental and physical health not only of children, but also, ultimately, their guardians.

## 5. Materials and Methods

This study was conducted with infants and children aged 2 months to 6 years and 7 months who attended licensed daycare centers, Tokyo Metropolitan certified daycare centers, hospital daycare centers, and corporate daycare centers operated by A-Childcare Co., Ltd. (Tokyo, Japan) (ACC) in 2013, 2014, and 2016. The significance of the sleep survey was explained to the guardians, and they were invited to participate voluntarily. Data on the sleep–wake state were obtained using a sleep log form, in which the cells representing every 30 min in 24 h were filled out to record sleep time continuously for two weeks. The sleep log forms were handed to the parents before starting the sleep measurement, and they were asked to fill in the form to record the sleep–wake state of their children at home for two weeks. The sleep–wake states of the children at nurseries were recorded by the staff and subsequently transcribed to their sleep log forms after the sleep measurement period.

The birth dates of the children and the names of the nurseries where they were attending were recorded as the basic data.

There were 7539 applicants, but records with insufficient information were excluded, therefore data from 4881 children were analyzed. The participants included 236 infants under 1 year of age, 1532 children aged 1 to 2 years, 1319 children aged 2 to 3 years, 842 children aged 3 to 4 years, 478 children aged 4 to 5 years, 355 children aged 5 to 6 years, and 119 children aged 6 to 7 years. This study was conducted in accordance with the Declaration of Helsinki and was approved by the ethical committees of ACC and H Prefectural Central Rehabilitation Hospital (#1532:2016/03/25). All parents of the children provided written informed consent before participating in the study. All analyses were performed in accordance with the Ethical Guidelines for Epidemiological Research in Japan.

[Period of Measurement]

The sleeping and waking states of the children were recorded for two weeks (14 days), from the 2nd to 15th of September 2013, from the 2nd to 15th of September 2014, and from the 5th to 18th of September 2016.

[Measurement Data][Definition of Analysis Items]

### 5.1. Night Bedtime

The night bedtime was defined as a time after 18:00 when the children were sleeping continuously for 1.5 h or more. However, if the bedtime was 00:30 or later, the earlier of the following two times listed below was recorded as the night bedtime:

(1) After 18:00, if the children slept for 0.5 h, woke up for 0.5 h or more, and then slept for 0.5 h or more after that, the time the children first fell asleep was considered the bedtime;
(2) The time when the children fell asleep after 18:00 and slept for at least one continuous hour.

If the children slept for only 30 min between 19:00 and 7:00 the following morning, the time at which the children fell asleep was considered to be the night bedtime. If the time at which the children fell asleep was later than midnight, 24 was added to the time. For example, if the time was 1:30, it was considered to be 25:30.

### 5.2. Morning Wake Time

The morning wake time was defined as a time after 4:00 in the morning when the children woke and continued to be awake for 2 h or longer. If the wake time by this definition was 7:30 or later, a time after 4:00 in the morning after which the children were waking routinely for 1.5 h or longer was recorded as the morning wake time. Furthermore, if the wake time by this definition was 10:30 or later, a time after 4:00 in the morning after which the children were waking continuously for 1 h or longer was reported as the morning wake time.

### 5.3. Total Sleep Duration

The sleeping or waking of the children was recorded across 48 points in time (with a 30 min interval between two adjacent points in time) from 0:00 to 23:30 on the day of measurement, and was denoted as *S*(*t*), where *S*(*t*) is 1 (sleeping) or 0 (waking). The total sleep duration is represented by the following formula, using these points in time:

$$Total\;sleep\;duration=\frac{\sum_{t=0:00}^{23:30}S(t)}{2}$$

### 5.4. Nocturnal Sleep Duration

The sleep time from the night bedtime to the morning wake time is the nocturnal sleep duration and is represented by the following formula:

$$Nocturnal\;sleep\;duration=\frac{\sum_{t=Night\;sleep-onset\;time}^{23:30}S(t)+\sum_{t=0:00}^{Morning\;wake\;time-30}S(t)}{2}$$

### 5.5. Daytime Sleep Duration

The daytime sleep duration is calculated by subtracting the nocturnal sleep duration from the total sleep duration.

[Statistical Analysis]

We analyzed the sleep logs of young children whose age at the time of the survey was recorded and whose data included analyzable sleep logs of at least eight weekdays and at least three weekend days.

The summary statistic values (number of cases, average, standard deviation (SD), first quartiles, medians, and third quartiles) of the night bedtime, morning wake time, total sleep duration, nocturnal sleep duration, and daytime sleep duration were calculated. Weekday sleep was defined as sleep from midnight (0 o’clock) on Monday to midnight (24 o’clock) on Friday, and weekend sleep was defined as sleep from midnight (0 o’clock) on Saturday to midnight (24 o’clock) on Sunday.

The statistical test was conducted using two-way analysis of variance (ANOVA), in which the factors were the day (weekdays and weekends) of the measurement, the age of the children, and their interaction. Multiplicity between time points was adjusted using Tukey–Kramer’s method. The trend in bedtime, waketime, and sleep duration across ages 0 to 6 years was analyzed using the Jonckheere–Terpstra trend test. R Version 4.0.2 (R Foundation for Statistical Computing, Vienna, Austria) was used for analysis with a significant difference of ±5%.

## Figures and Tables

**Table 1:** Shift from weekday sleep categories to weekend sleep categories.

		Weekends				
	Class	1. Group with Sufficient Sleep	2. Group with Excessive Sleep	3. Group with Sleep Deprivation	4. Group with Delayed Sleep Phase	Total
Weekdays	1. Group with sufficient sleep	1342 (80.3%)	330 (19.7%)	0	0	1672
	2. Group with excessive sleep	17 (14.9%)	97 (85.1%)	0	0	114
	3. Group with sleep deprivation	0	0	1157 (51.2%)	1103 (48.8%)	2260
	4. Group with delayed sleep phase	0	0	81 (9.7%)	754 (90.3%)	835
	Total	1359	427	1238	1857	4881

## Data Availability

The data presented in this study are available upon request from the corresponding author, due to privacy.

## Abbreviation

NBSD	Nighttime basic sleep duration
